# Remote Monitoring of Bioelectrical Impedance in Patients With Breast Cancer–Related Lymphedema: 1-Year Pilot Longitudinal Study

**DOI:** 10.2196/86624

**Published:** 2026-05-05

**Authors:** Lori Bravi, Cheri Teranishi-Hashimoto, Elena A C MacDonald, Dayna Huntley, Samman Shahpar, Jami Fukui, Monica Padilla, Yoomi Heo, Linda A Koehler

**Affiliations:** 1 Shirley Ryan AbilityLab, Chicago, IL, United States; 2 Nuuanu Clinic, Rehabilitation Hospital of the Pacific, Honolulu, HI, United States; 3 Department of Family Medicine and Community Health, Division of Physical Therapy and Rehabilitation Science, University of Minnesota, Minneapolis, MN, United States; 4 Tennessee Oncology-Nashville Breast Center, Nashville, TN, United States; 5 Cancer Rehabilitation Program, Shirley Ryan AbilityLab, Chicago, IL, United States; 6 Cancer Biology-Translational and Clinical Research, University of Hawaii Cancer Center, Manoa, HI, United States; 7 Clinical Research Department, InBody BWA, Trooper, PA, United States; 8 Clinical Research Department, InBody Co, Ltd., InBody Bldg, 625, Eonju-ro, Gangnam-Gu, Seoul, 06106, Republic of Korea, 82 1026913661; 9 Masonic Cancer Center and Department of Family Medicine and Community Health, Division of Physical Therapy and Rehabilitation Science, University of Minnesota, Minneapolis, MN, United States

**Keywords:** bioelectrical impedance analysis, breast cancer-related lymphedema, ECW/TBW ratio, longitudinal study, patient-led monitoring, diurnal variation

## Abstract

**Background:**

Breast cancer–related lymphedema (BCRL) is a chronic complication that impairs quality of life through persistent fluid accumulation. While clinical guidelines recommend longitudinal surveillance, implementation is often limited by the logistical challenges of frequent in-clinic visits. Bioelectrical impedance analysis (BIA), specifically the segmental extracellular water–to–total body water (ECW/TBW) ratio, offers a noninvasive method for tracking fluid status. However, the technical agreement between in-clinic and patient-led home-based BIA systems, as well as the feasibility of long-term self-monitoring in real-world settings, remains to be fully established.

**Objective:**

Our primary objective was to evaluate the agreement between in-clinic and home-based BIA systems for key body composition and fluid-related parameters. Our secondary objective was to characterize longitudinal fluid patterns and diurnal variations in ECW/TBW ratios to assess the feasibility of home-based monitoring.

**Methods:**

This prospective, patient-driven, 12-month observational study enrolled breast cancer survivors at risk for lymphedema. Agreement between the in-clinic BIA system (InBody 770) and the home-based device (BWA ON) was assessed using Bland-Altman analysis, intraclass correlation coefficients (ICCs), and the Lin concordance correlation coefficient (CCC). Longitudinal home-based ECW/TBW measurements were analyzed using linear mixed-effects models to evaluate diurnal differences (before-noon vs after-noon) across groups defined by limb dominance and BCRL status (International Society of Lymphology [ISL] stage 0 vs stage 1).

**Results:**

Over 12 months, ECW/TBW ratios measured by the home-based device demonstrated strong agreement with in-clinic measurements, showing minimal bias and high ICC/CCC values. Longitudinal analysis revealed that ECW/TBW changes did not follow uniform patterns within ISL stage categories, showing substantial physiological heterogeneity even among clinically stable groups. Diurnal analysis identified a small but statistically significant decrease in ECW/TBW ratios in the afternoon (*P*<.001). The magnitude of this decrease differed by limb dominance and BCRL status, with the most pronounced reduction observed in participants whose dominant arm was affected and who had a history of stage 1 lymphedema. ECW/TBW variability was driven more by within-individual factors (eg, measurement timing) than by between-individual differences.

**Conclusions:**

Home-based segmental bioimpedance provides reliable longitudinal data and reveals granular fluid patterns not captured by conventional ISL staging alone. The significant impact of diurnal variation, particularly in relation to limb dominance, underscores the need for standardizing measurement protocols. Standardizing home-based measurements to a fixed monitoring time can minimize physiological noise and enhance the interpretability of long-term self-monitoring strategies for breast cancer survivors.

## Introduction

Breast cancer is the most commonly diagnosed cancer and the leading cause of cancer-related death in women, followed by lung cancer [1]. Breast cancer mortality is decreasing due to improved diagnostics and treatments. As survival is increased, the long-term sequelae of treatment and impaired quality of life become relevant [2]. Because of improvements in cancer diagnostic modalities, surgical technology, and targeted treatment, breast cancer mortality has been continuously decreasing during the past few decades, and the number of long-term cancer survivors is consequently continuing to increase. However, many breast cancer survivors develop negative sequelae, such as lymphedema, neuropathy, shoulder morbidity, depression, and anxiety [3].

Lymphedema is a chronic condition characterized by abnormal lymph fluid accumulation and is broadly classified as primary or secondary. Primary lymphedema results from developmental abnormalities of the lymphatic system, whereas secondary lymphedema is caused by acquired damage to lymphatic vessels due to trauma, tumor, surgery, infection, or cancer treatment. In transitional countries, secondary lymphedema is most commonly associated with infectious or parasitic diseases affecting lymphatic channels, while in high-income countries it most frequently occurs following lymph node dissection for cancer management. Clinically, lymphedema is characterized by chronic swelling, localized pain, atrophic skin changes, localized fluid imbalance, and recurrent secondary infections, regardless of etiology. Beyond physical symptoms, one of the most debilitating aspects of lymphedema is visible limb disfigurement, which is strongly associated with psychological morbidity and impaired quality of life [4,5].

Breast cancer–related lymphedema (BCRL) is the most common form of secondary lymphedema, reflecting the high global prevalence of breast cancer among women. BCRL affects approximately 3‐5 million individuals worldwide, with an estimated 20,000 new cases annually in the United States, and reported incident rates range widely from 2% to 77% depending on the extent of local-regional and systemic therapies [6]. BCRL arises from disruption of the lymphatic system during breast cancer treatment, particularly following axillary lymph node dissection (ALND), radiation therapy, and systemic chemotherapy [1,2,7-9]. Historically, BCRL incidence has reached 20%‐40% in patients undergoing ALND [5-11]. Established risk factors include the number of lymph nodes removed, exposure to chemotherapy and radiation, elevated BMI, and patient comorbidities [1,3,5,12,13]. Given the high incidence and chronic nature of BCRL, continuous and accessible monitoring of limb fluid status is essential for maintaining the quality of life in this growing survivor population.

Early lymphedema, defined as subclinical disease or stage 1 according to the International Society of Lymphology (ISL) classification, has been shown to be potentially reversible when identified and managed promptly [3,14-17]. In contrast, progression to stage II or III is associated with chronic tissue changes, including fibrosis, rendering the condition reducible but not reversible and less responsive to intervention [7,8]. Accordingly, early identification of BCRL is widely regarded as a critical determinant of long-term outcomes.

Major oncology, survivorship, rehabilitation, and lymphology societies consistently recognized BCRL as a common and potentially lifelong complication resulting from lymphatic system damage following breast cancer treatment, particularly after axillary lymph node surgery [6,8]. The National Comprehensive Cancer Network’s breast cancer and survivorship guidelines emphasize that longitudinal surveillance and individualized baseline assessments are essential for optimal LE management and recommend pretreatment bilateral limb measurements to establish an individualized baseline in patients with treatment-related or personal risk factors [16,18,19]. These guidelines further note that while BCRL most commonly develops within the first 3 years after treatment, it may occur at any point during survivorship, and that early-stage disease (stages 0‐1) is potentially reversible, whereas later-stage disease is not [8].

In parallel, the American Society of Clinical Oncology and the American Cancer Society highlight the importance of survivor education focused on risk reduction and recommend prompt referral for patients presenting with symptoms or clinical signs suggestive of lymphedema [5]. Rehabilitation- and lymphology-focused organizations further advocate for objective surveillance strategies, with the American Physical Therapy Association recommending the use of bioimpedance analysis to detect subclinical or early-stage lymphedema [7], and the Australasian Lymphology Association advising routine preoperative assessment using limb circumference measurements and/or bioimpedance spectroscopy, followed by risk-adapted longitudinal monitoring [10]. Similarly, the British Lymphology Society underscores the need for structured patient education prior to cancer treatment, clear referral pathways, and access to designated health care professionals to facilitate early intervention and psychosocial support [9]. Collectively, these recommendations support a proactive and standardized approach to BCRL management that integrates baseline assessment, ongoing surveillance, patient education, and timely referral to specialized lymphedema services.

Despite these consensus recommendations, diagnosing preclinical or subclinical lymphedema remains challenging and requires baseline assessment and surveillance at standardized intervals [9,14-17]. Clinically, BCRL has been defined as a ≥2-cm increase in limb circumference, a ≥200-mL increase in limb volume, or a 5%‐10% change in limb volume compared to the unaffected arm [9-13]. However, there is no single diagnostic criterion with high sensitivity and specificity for lymphedema [8]. Volume-based methods, including tape measurement, water displacement, and perometry, do not differentiate among bone, muscle, fat, and lymphatic or extracellular fluid and are therefore limited in their ability to capture dynamic or transient fluid fluctuations occurring between clinical visits [20,21].

Although physical examination and clinical history remain central to diagnosis, early swelling may be transient; one study reported that approximately one-third of initial swelling episodes resolve spontaneously, further complicating early identification [22]. Given that effective intervention is most successful in early stages, accurate and timely detection of subclinical disease is essential to prevent progression [23].

Bioimpedance techniques provide a fast, noninvasive approach to assessing body water distribution by applying electrical currents at multiple frequencies [3]. At low frequencies, electrical current is restricted to the extracellular compartment and inversely reflects extracellular water (ECW), whereas at higher frequencies it traverses cell membranes, allowing estimation of total body water (TBW) [3]. Because lymphedema is fundamentally characterized by ECW accumulation, bioimpedance is particularly well suited for its detection [4,14,24-29]. Deviations from an individual’s baseline bioimpedance values may indicate subclinical lymphedema, providing objective quantification of subtle fluid status that may not be captured by volume-based methods alone, thereby supporting more granular longitudinal tracking [4,14,24-28,30].

Segmental bioelectrical impedance analysis (BIA) further enhances clinical utility by dividing the body into five segments (both arms, both legs, and the trunk) and simultaneously measuring impedance in each segment [31-34]. Segmental ECW/TBW ratios are used to assess fluid imbalance, with values between 0.360 and 0.390 considered physiologically normal and values of >0.390 suggestive of abnormal ECW accumulation [31,35,36]. Because each limb is referenced to its own TBW rather than a contralateral comparison, this approach allows assessment of both unilateral and bilateral lymphedema, objective quantification of limb fluid status, and longitudinal monitoring of treatment response [31-34].

Accordingly, this study was designed as a prospective, patient-driven, real-world observational study reflecting routine clinical practice. In standard post–breast cancer care, lymphedema surveillance and preventive management are typically conducted in rehabilitation medicine clinics at approximately 3-month intervals. While these scheduled, hospital-based assessments provide structured clinical oversight, they are limited in their ability to capture dynamic changes occurring in patients’ daily lives between visits.

To address this gap, this study aimed to evaluate how daily, home-based bioelectrical impedance measurements, obtained under real-world conditions, reflect physiological fluid trends. By prospectively collecting patient-generated data, this study sought to characterize the consistency of home-based BIA and analyze how diurnal and behavioral variability influence these metrics. This approach aims to provide evidence for the feasibility of home-based monitoring as a reliable, guideline-concordant strategy that complements periodic in-clinic assessments by providing high-frequency, longitudinal data.

## Methods

### Participants

**Figure 1.**
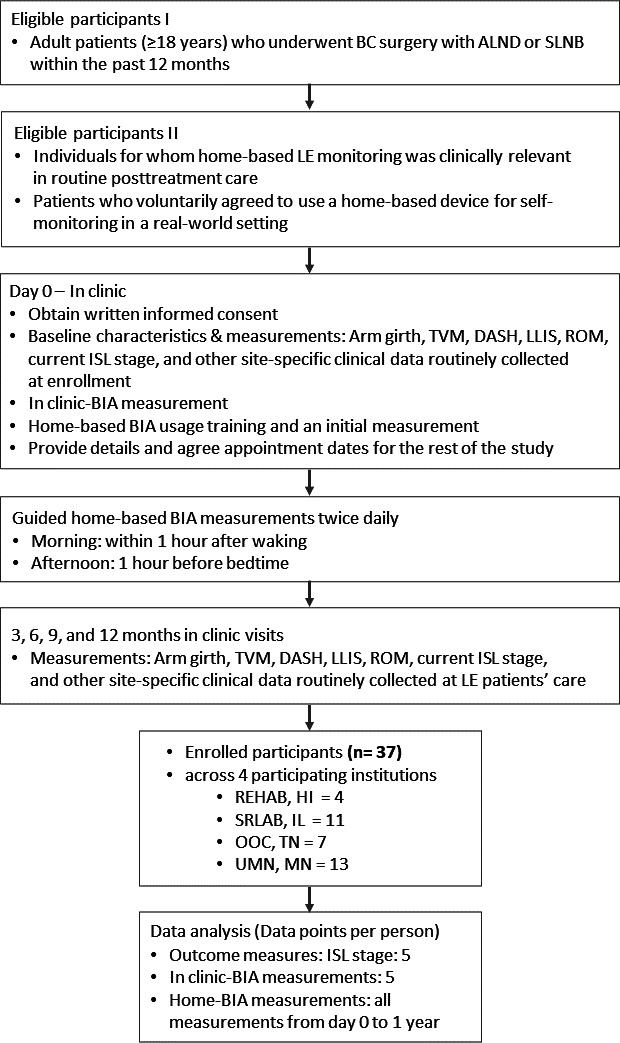
Participant flow and longitudinal monitoring protocol. Participants were enrolled from four institutions over a 29-month period, with each individual followed for a 12-month monitoring period. Baseline in-clinic assessments included site-specific clinical data and bioelectrical impedance analysis (BIA) measurements to establish individual baselines. After receiving standardized training, participants performed patient-led BIA measurements at home, complemented by in-clinic follow-up visits every 3 months for continuous lymphedema surveillance. ALND: axillary lymph node dissection; BC: Breast cancer; DASH: Disabilities of the Arm, Shoulder, and Hand; ISL: International Society of Lymphology; LE: lymphedema; LLIS: Lymphedema Life Impact Scale; OOC: One Oncology Center; REHAB: REHAB Hospital of the Pacific; ROM: range of motion; SLNB: sentinel lymph node biopsy; SRLAB: Shirly Ryan Laboratory; TVM: tape volume measurement; UMN: University of Minnesota.

This study was a multicenter study conducted across four tertiary hospitals. This involved five in-person clinic visits (baseline, 3, 6, 9, and 12 months) and daily home-based body composition measurements over 1 year. Eligible participants were adult patients (aged 18 years or older) who underwent breast cancer surgery an axillary lymph node dissection or sentinel lymph node biopsy and were therefore considered at risk of developing lymphedema to monitor longitudinal fluid changes. Prophylactic mastectomy and reconstruction were permitted. Participants were excluded if they had a bilateral cancer diagnosis, were pregnant, had an infection (ie, cellulitis), or had an implanted electrical medical device (ie, cardiac pacemaker). The participants were in the self-care phase of treatment. Enrollment took place between February 2023 and July 2024 at four institutions: the REHAB Hospital of the Pacific (Honolulu, Hawai’i), Shirley Ryan AbilityLab (Chicago, Illinois), One Oncology Tennessee (Nashville, Tennessee), and the University of Minnesota (Minneapolis, Minnesota). Recruitment was planned to be evenly distributed across the four regions; however, the number of enrolled participants varied by site during the recruitment period (see Figure 1).

### Ethical Considerations

This prospective, patient-driven, real-world observational study was reviewed and approved by the institutional review boards of all participating sites. Ethical approvals were obtained from the University of Hawai‘i Institutional Review Board (REHAB Hospital of the Pacific; IRB No. IRB2022-00055), the Northwestern University Institutional Review Board (Shirley Ryan AbilityLab; IRB No. STU00216604), the BRANY Institutional Review Board (One Oncology Tennessee; IRB No. 24-08-131-652), and the University of Minnesota Institutional Review Board (IRB No. STUDY00021973). The study was conducted in accordance with the principles of the Declaration of Helsinki and all applicable human research regulations. Written informed consent was obtained from all participants prior to their participation in the study.

### In-Clinic and Home-Based Monitoring Protocol

Clinical assessments were conducted as part of routine post–breast cancer care at each participating site. At enrollment, participants underwent standard clinical evaluations to characterize their baseline fluid status, including determination of the ISL stage of lymphedema. In-clinic BIA measurements were performed. All clinical data collected during these visits reflected information routinely obtained during standard patient care and were not collected solely for research purposes. Participants enrolled in the study received standardized instruction on the use of the home-based BIA device, and the initial home-based BIA measurement was performed in the clinic at the time of training. After returning home, participants were instructed to perform self-administered BIA measurements twice daily: once in the morning within 1 hour after waking as part of a consistent daily routine and once in the evening within 1 hour before bedtime to capture diurnal variations within a consistent daily routine. Adherence to this long-term self-monitoring schedule was encouraged to reflect real-world engagement without clinical supervision (see Figure 1).

Consistent with routine LE surveillance practices, participants attended in-clinic follow-up visits every 3 months over a 12-month period (at months 3, 6, 9, and 12). During these visits, lymphedema status was reassessed, and participants received guidance on self-management based on clinical findings, while continuing home-based BIA measurements throughout the monitoring period. Participants were referred for further treatment as clinically indicated based on in-clinic evaluation findings, including range-of-motion deficits, lymphedema symptoms, tissue or integumentary issues, and reduced function or mobility.

### Conventional Criteria for BCRL

Conventional criteria were used to define the onset and stage of BCRL across participating institutions. In routine clinical practice, BCRL onset in the at-risk arm was assessed using circumferential tape measurements and volume-based criteria comparing the ipsilateral and contralateral upper extremities. Although site-specific clinical assessments were conducted according to local practice at each institution, lymphedema staging in this study was standardized using the widely accepted ISL classification system, including subclinical lymphedema (stage 0) and stages 1‐3. Stage 0 was defined as a latent or subclinical condition characterized by impaired lymphatic transport without clinically apparent swelling, whereas stages 1 through 3 were defined according to ISL criteria based on the presence and progression of clinically observable edema and tissue changes. In this study, particular emphasis was placed on monitoring participants at stages 0 and 1, as these stages represent the subtle physiological fluid fluctuations that home-based BIA is designed to track over time. Throughout the study period in the clinical setting, all participants were managed while remaining asymptomatic or classified as stage 0 or 1, and no cases of stage 2 or 3 lymphedema were observed.

### BIA Measurements

BIA measurements were obtained using an in-clinic device (InBody 770 [IB770]; InBody Co, Ltd) and a home-based device (BWA ON; InBody Co, Ltd). IB770 is a body composition analyzer widely used in body composition research and provides standard BIA-derived parameters, including fat-free mass, soft lean mass, body fat mass, and ECW/TBW ratios, with the ability to measure segmental ECW/TBW values. IB770 measurements were used as an in-clinic reference to evaluate the consistency of this metric across devices. Both systems are multifrequency bioimpedance analyzers designed to support region-specific assessment relevant to lymphedema surveillance. IB770 was used for standardized in-clinic assessments as part of routine clinical care, whereas BWA ON was used for self-administered home measurements, with results displayed via a dedicated mobile app on the participant’s personal smartphone. Measurement data generated by BWA ON were assigned a unique participant-specific identifier and encrypted prior to transmission. Encrypted data were securely transferred through a cloud-based system to the participant’s affiliated institution, where access was restricted to authorized health care professionals and researchers for clinical monitoring and research purposes. No directly identifiable personal information was stored or displayed within the app beyond what was required for secure data linkage.

### Evaluation Criteria

Evaluation criteria were defined a priori to distinguish device-level agreement from longitudinal home-based measurement characteristics. The primary outcome was the agreement between the home-based BIA device (BWA ON) and the in-clinic reference device (IB770) to assess whether the home-based system provided measurements suitable for clinical monitoring. This comparison was based on paired measurements obtained during the initial in-clinic visit, at which both devices were used under standardized conditions. The secondary outcome focused on within-day variation in home-based BIA measurements obtained using BWA ON during daily self-monitoring. Specifically, differences in segmental ECW/TBW ratios between morning (before-noon) and afternoon (after-noon) measurements were evaluated to identify inherent diurnal variations that could influence the interpretation of long-term trends. In addition, analyses incorporated limb dominance as a clinically relevant factor, reflecting conventional bioimpedance interpretation practices in patients with lymphedema, in which dominant and nondominant limbs may exhibit systematic physiological differences. Accordingly, secondary analyses examined whether diurnal changes in segmental ECW/TBW differed depending on whether the affected (at-risk) limb corresponded to the dominant or nondominant upper extremity. The feasibility of the monitoring system was evaluated based on participant adherence to the twice-daily measurement protocol.

### Data Analysis

The analytic dataset consisted of repeated measurements of clinical lymphedema status (ISL stage), in-clinic BIA data (IB770), and longitudinal home-based BIA data (BWA ON). To evaluate agreement between the in-clinic and home-based BIA devices, the Lin concordance correlation coefficient (CCC) was used as the primary measure of agreement, supplemented by Pearson correlation coefficients and intraclass correlation coefficients (ICCs; two-way mixed-effects model with absolute agreement). Bland-Altman analysis was additionally performed to assess mean differences and 95% limits of agreement.

To illustrate the heterogeneity of home-based BIA change patterns within the ISL stage framework, grouped bar charts were used to visualize coexisting directional changes across stage transition categories. For diurnal variation analyses, paired *t* tests were conducted for only days where both morning (before-noon) and afternoon (after-noon) measurements were available to compare mean ECW/TBW differences at the individual level.

To account for the hierarchical and repeated-measures structure of the full longitudinal dataset, linear mixed-effects models (LMMs) were applied. In these models, measurement timing (before-noon vs after-noon) and limb status (affected vs unaffected) were treated as fixed effects, while participant IDs were included as random effects to control for within-individual correlation. Data distributions were examined and visualized using box plots and grouped bar charts to facilitate comparison across measurement conditions. Statistical significance was defined as a two-tailed *P* value of <.05. All statistical analyses were conducted using Python (version 3.13).

## Results

### Participant Demographics

A total of 37 female patients were initially enrolled in the study. However, two individuals who self-identified as ambidextrous were excluded from the analysis, as limb dominance was one of the key analytical variables in this study. For outcome analyses, participants were categorized based on ISL staging; however, stage 0 was further stratified to reflect longitudinal clinical status during the monitoring period. Specifically, participants who remained consistently within stage 0 throughout follow-up were distinguished from those who met the criteria for stage 0 but experienced transient fluctuations in clinical status during the study period. This stratification was applied to account for the known heterogeneity of subclinical lymphedema and to better capture differences in daily ECW distribution patterns observed through home-based bioimpedance monitoring.

At the time of program enrollment, all cases were unilateral with respect to the affected arm (right or left). In participants who had previously undergone bilateral mastectomy, those with a clearly defined affected and unaffected arm based on clinical management were included and monitored accordingly. For the final analysis, participants were categorized according to whether the affected arm was the dominant or nondominant limb, as limb dominance was a key variable of interest in this study. Baseline participant characteristics were therefore summarized by affected-side dominance (dominant vs nondominant). No statistically significant differences were observed across the four resulting groups with respect to age, body weight, or BMI.

**Table 1.** Participants’ arm dominance and lymphedema status are shown along with demographic information (n=35).

Column headers	Dominant affected: stage 1	Dominant affected: stage 0	Nondominant affected: stage 1	Nondominant affected: stage 0
Participants, n	4	11	7	13
Age (years), mean (SD), range	49.75 (10.50), 41‐62	51.45 (14.43), 34‐82	57.29 (8.48), 45‐68	51.69 (9.13), 39‐66
Weight (kg), mean (SD), range	77.70 (21.26), 51.03‐102.78	74.51 (19.07), 41.91‐104.50	77.52 (7.65), 69.35‐92.40	69.91 (10.07), 50.53‐86.20
BMI (kg/m^2^), mean (SD), range	28.93 (7.91), 18.72‐37.71	27.52 (6.74), 17.81‐38.43	28.89 (2.51), 24.00‐31.81	26.13 (3.94), 20.38‐33.46
Lesion side (R:L)^a^	4:0	10:1	0:6^b^	2:11
Surgery type (BM:UM:LP)^c^	0:1:1	2:1:1	3:0:4	1:7:5
Lymph-node procedure[Table-fn T1_FN4] (ALND:SLND:SLNB)^d^	1:0:3	1:4:6	2:0:5	2:3:8

a R: right; L: left.

b One participant has no record of her lesion side.

c BM: bilateral mastectomy; UM: unilateral mastectomy; LP: lumpectomy.

d ALND: axillary lymph node dissection; SLND: sentinel lymph node dissection; SLNB: sentinel lymph node biopsy.

### Patient-Led Home-Based Measurement Adherence

Adherence to the patient-led home-based BIA protocol is summarized in Table 2. Over the 12-month monitoring period, 35 participants across four geographically diverse sites (Honolulu, Hawai’i; Chicago, Illinois; Nashville, Tennessee; and Minneapolis, Minnesota) proactively contributed a total of 16,425 measurements using the BWA ON device. This high volume of data yielded an overall adherence rate of 64.3% relative to the recommended twice-daily measurement schedule.

**Table 2.** Patient engagement and adherence to the home-based bioelectrical impedance analysis measurement protocol over 12 months. The table summarizes the number of enrolled participants per site, the total volume of patient-led BWA ON measurements, overall adherence relative to the recommended twice-daily schedule, and the diurnal distribution (before vs after noon) of the recorded data.

Institution (site)	Number of participants, n	Total BWA ON tests, n	Adherence (%)	Before-noon tests, n (%)	After-noon tests, n (%)
1 (Honolulu, Hawai’i)	9	4325	65.8	2522 (58)	1803 (42)
2 (Chicago, Illinois)	6	2142	48.9	1225 (57)	917 (43)
3 (Nashville, Tennessee)	9	3662	55.7	1949 (53)	1713 (47)
4 (Minneapolis, Minnesota)	11	6296	78.4	3502 (56)	2794 (44)
Total	35	16,425	64.3	9198 (56)	7227 (44)

Notably, while adherence varied across sites—ranging from 48.9% (Chicago, Illinois) to 78.4% (Minneapolis, Minnesota)—all sites generated sufficiently dense longitudinal data to support the feasibility of extended, patient-driven monitoring in a real-world setting. Furthermore, as described in Table 2, the diurnal distribution of measurements was remarkably consistent across all regions. Before-noon measurements accounted for 56% of the total dataset, while after-noon measurements comprised 44%. This balanced distribution indicates that participants maintained a stable measurement routine regardless of their clinical site, ensuring sustained engagement with both daily measurement windows throughout the monitoring period. This consistency confirms that the home-based system is practical for capturing both morning and afternoon fluid status without clinical supervision.

### Agreement Between in-Clinic and Home-Based BIA

To evaluate the agreement between the in-clinic BIA system (IB770) and the home-based device (BWA ON), paired measurements obtained at the enrollment visit were analyzed. Correlation analyses demonstrated strong positive associations between the two devices across all parameters, with correlation coefficients exceeding 0.99 for weight and percent body fat, and 0.86 for segmental ECW/TBW ratios. Of the 35 participants, two were excluded from the agreement analysis due to significant data entry errors in height (unit confusion between feet and inches), which resulted in physiologically implausible outliers. Therefore, the final agreement analysis was conducted with 33 participants.

**Figure 2.**
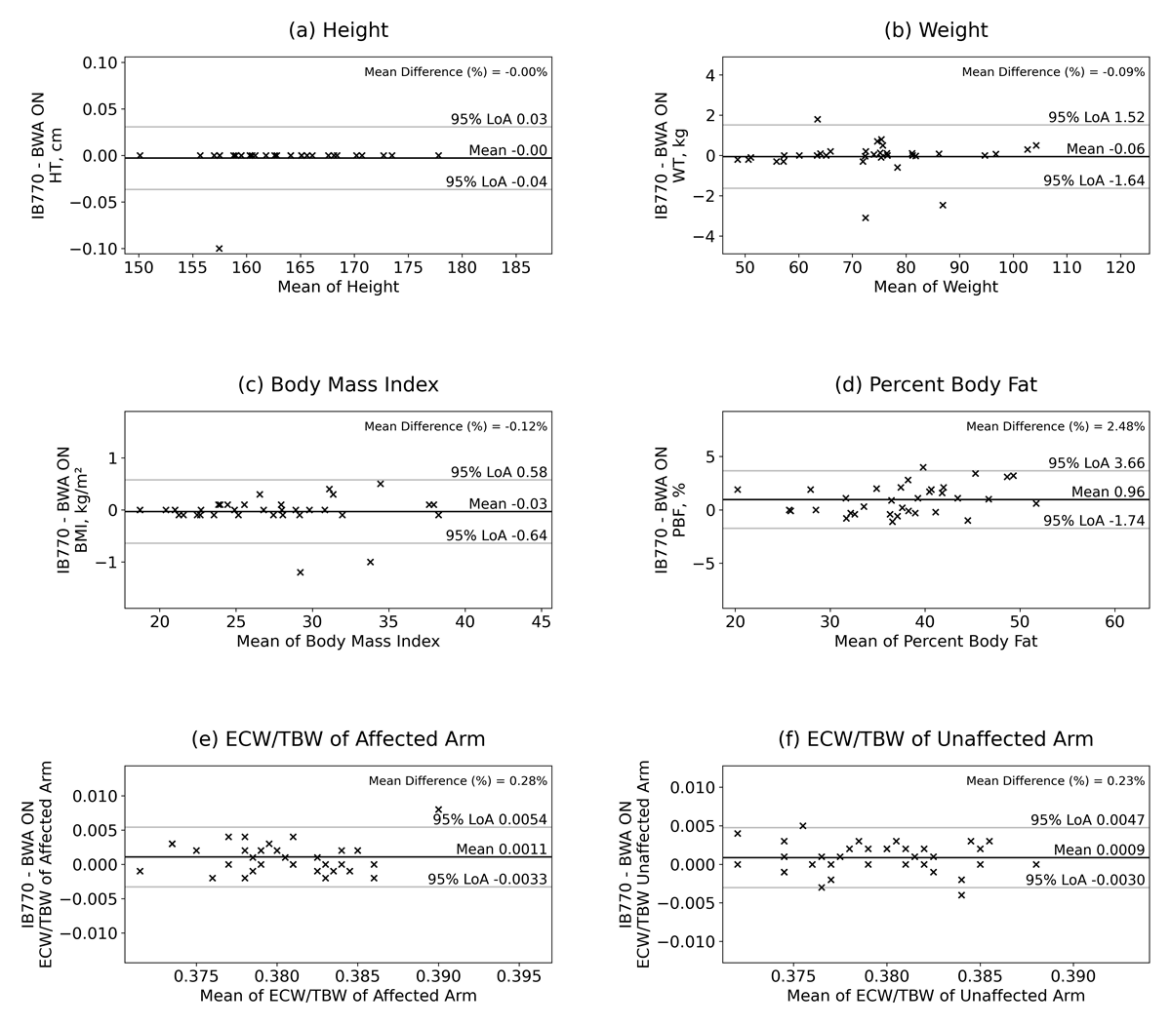
Bland-altman plots illustrating the agreement between home-based (BWA ON) and in-clinical (InBody 770) bioelectrical impedance analysis measurements. Panels represent (A) height, (B) weight, (C) BMI, (D) percent body fat (PBF), (E) ECW/TBW ratio of the affected arm, and (F) ECW/TBW ratio of the unaffected arm. The solid line indicates the mean bias, and the dashed lines represent the 95% limits of agreement (±1.96 SD). ECW: extracellular water; TBW: total body water.

Bland-Altman analyses showed minimal mean bias (0.001) for ECW/TBW measurements in both the affected and unaffected arms, with narrow 95% limits of agreement (Figure 2). ICC and CCC values further supported substantial agreement between the systems. Collectively, these findings indicate that home-based BIA measurements obtained using the BWA ON device are highly consistent with professional in-clinic standards. This high level of technical agreement supports the use of the home-based system as a reliable tool for high-frequency longitudinal monitoring, providing data quality comparable to standardized clinical assessments.

**Table 3.** Agreement between home-based (BWA ON) and in-clinic (InBody 770) bioelectrical impedance analysis measurement at enrollment (n=33). Agreement was evaluated using Bland–Altman analysis, intraclass correlation coefficients (ICCs, two-way mixed-effects model, and absolute agreement), and the Lin concordance correlation coefficient (CCC). Results are presented for anthropometric and body composition parameters, including weight, BMI, percent body fat (PBF), and segmental extracellular water–to–segmental total body water (ECW/TBW) ratios for the affected and unaffected upper extremities. Reported metrics include mean bias, SD of the error, 95% limits of agreement (LoA), proportion of measurements outside the LoA, ICC, *P* values, and CCC. Two participants were excluded from this analysis due to erroneous height input during the initial home-based measurement, resulting in extreme outliers.

Output	Mean error (bias)	SD of error (SD)	LoA lower limits	LoA upper limits	Proportion outside LoA (%)	ICC (3,1)	*P* value	CCC
Height	0	0.02	0	0	2.94	1	<.001	0.901
Weight	–0.1	0.8	–1.6	1.5	8.82	0.998	<.001	0.998
BMI	0	0.31	–0.6	0.6	5.88	0.998	<.001	0.974
PBF	1	1.38	–1.7	3.7	2.94	0.981	<.001	0.948
ECW/TBW affected arm	0.001	0.0022	–0.003	0.005	2.94	0.868	<.001	0.849
ECW/TBW unaffected arm	0.001	0.002	–0.003	0.005	5.88	0.889	<.001	0.886

### Longitudinal Changes in ISL Stage and Bioimpedance Parameters Over 12 Months

To evaluate longitudinal trends in ECW distribution over the 12-month monitoring period, participants’ clinical lymphedema status was defined using ISL stage assessments obtained during five scheduled in-clinic visits for lymphedema management. These visits, occurring at approximately 3-month intervals, served as the reference standard for evaluating changes in edema status over time. Based on ISL staging across consecutive visits, temporal changes were categorized into four clinical transition patterns: worsening (stages 0-1), improvement (stages 1-0), stable low-risk status (persistent stage 0), and stable persistent status (persistent stage 1). These categories were designed to capture the full spectrum of LE progression and regression, as well as periods of clinical stability.

Corresponding to each clinical visit interval, home-based bioimpedance data were analyzed to determine whether segmental ECW trends measured by the BWA ON system exhibited concordant directional changes. The ECW/TBW ratio was used as the primary bioimpedance-derived indicator, with values exceeding 0.390 considered indicative of abnormal extracellular fluid accumulation. For each intervisit interval, changes in the ECW/TBW ratio were classified according to directional trends relative to the previous interval: a “decrease” reflected a shift toward edema improvement, an “increase” indicated a shift toward worsening, and minimal change was defined as “stable” status maintenance.

**Figure 3.**
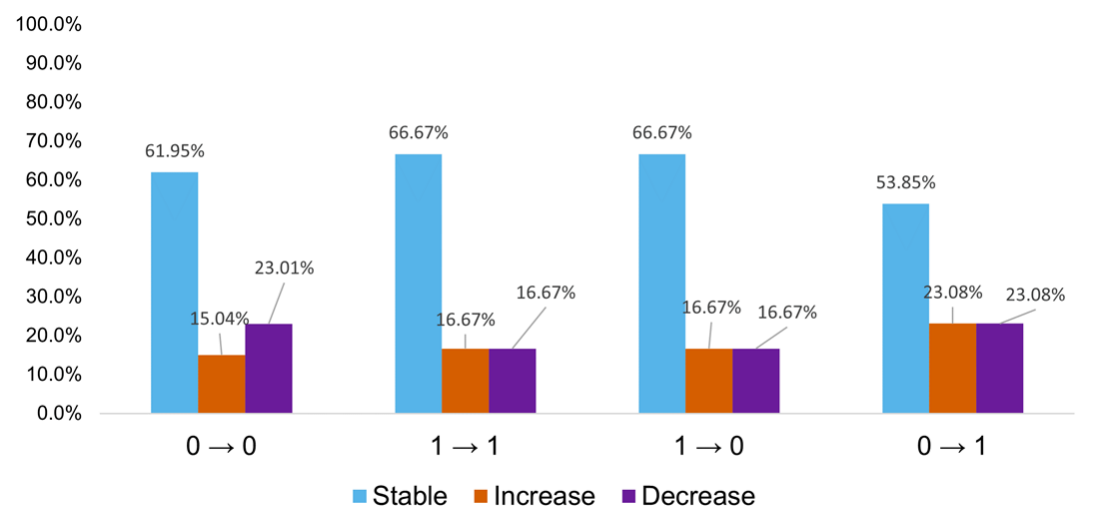
Divergent ECW/TBW trends within the ISL stage framework. A 12-month longitudinal analysis. Grouped bar charts illustrate the inherent heterogeneity of home-based BIA directional patterns across clinical stage transitions. Notably, substantial variability in ECW/TBW behavior was observed even within clinically stable categories (0→0 and 1→1), indicating that segmental bioimpedance captures subtle physiological fluctuations not reflected by conventional ISL staging. These distinct distributions across clinical risk contexts highlight the increased granularity of high-frequency, patient-led monitoring compared to intermittent clinical assessments. BIA: bioelectrical impedance analysis; ECW: extracellular water; ISL: International Society of Lymphology; TBW: total body water.

These bioimpedance-based transition patterns were examined in relation to the corresponding ISL stage transitions to assess whether home-based ECW/TBW trends reflected clinically observed changes. As illustrated in Figure 3, the results demonstrated that ISL stage classification alone does not fully capture the underlying heterogeneity of BWA ON change patterns. Across all stage transition categories, BWA ON measurements did not follow a uniform directional pattern. Notably, heterogeneous distributions were observed even within clinically stable groups (0→0 and 1→1), revealing substantial variability in bioimpedance behavior that remained undetected within the same clinical stage framework. These distinct distributions across clinical risk contexts suggest that patient-led monitoring provides a more granular level of physiological information than intermittent clinical staging.

### Impact of Measurement Timing, Limb Dominance, and BCRL Status on Diurnal ECW/TBW Patterns

This analysis investigated whether ECW/TBW ratios obtained in a home-based environment demonstrated systematic variation according to measurement time within a day. Furthermore, we assessed how these ratios were influenced by the interaction between measurement timing, limb dominance (dominant vs nondominant), and BCRL status (stage 0 vs stage 1). These associations were evaluated using LMMs to account for the hierarchical nature of repeated measurements within participants.

Initially, we examined whether the ECW/TBW ratio of the affected arm varied according to measurement time (before-noon vs after-noon) and whether it differed depending on whether the affected arm corresponded to the dominant or nondominant limb. The results of the stepwise LMM are presented in Table 4. The estimated mean ECW/TBW ratio of the affected arm at the before-noon time point was 0.380 (intercept coefficient, *P*<.001). Compared to before-noon measurements, the after-noon ECW/TBW ratio was significantly decreased by 0.001 (*P*<.001). The between-group variance was minimal (group variance=0.000; *P*<.001), indicating that variability in the ECW/TBW ratio was primarily associated with measurement timing rather than interindividual differences.

**Table 4.** Linear mixed-effects model evaluating diurnal variation in the extracellular water (ECW)/total body water (TBW) ratios in the affected arm. The table presents the fixed effects of measurement timing (before-noon vs after-noon) on the segmental ECW/TBW ratio of the affected limb. The model demonstrates that systematic diurnal fluctuations significantly contribute to the variability of bioimpedance metrics, independent of interindividual differences. Statistical significance was defined as *P*<.05.

Fixed effect	Estimate	95% CI	*P* value
Fixed effects			
Intercept (before-noon)^a^	0.380	0.378 to 0.381	<.001
Diurnal change (after-noon)^b^	–0.001	–0.001 to –0.001	<.001
Random effect			
Participant (group variance)^c^	0.000	—^d^	<.001

a Intercept represents the baseline mean ECW/TBW ratio of all affected limbs measured during the before-noon period.

b Diurnal change indicates the estimated mean reduction in the ECW/TBW ratio during the after-noon period compared to the before-noon reference.

c Group variance represents the between-individual variance; a near-zero value indicates that variability is primarily driven by within-individual factors (measurement timing).

d Not applicable.

Based on the premise that limb dominance influences bioimpedance interpretation, we further stratified participants into four groups. The reference group consisted of participants whose dominant arm was affected and who consistently maintained stage 0 throughout follow-up (Same & non-BCRL). In this baseline group, the mean ECW/TBW ratio was estimated at 0.378 (*P*<.001), with a modest but significant afternoon decrease of 0.001 (*P*<.001). The results of this comprehensive interaction analysis are summarized in Table 5.

**Table 5.** Linear mixed-effects model of diurnal extracellular water (ECW)/total body water (TBW) ratio variations by limb dominance and breast cancer–related lymphedema (BCRL) status. The table illustrates the estimated differences in affected-arm ECW/TBW ratios between before-noon and after-noon measurements, stratified by limb dominance (“same” vs “different”) and clinical BCRL status (“BCRL” vs “non-BCRL”). The “same & non-BCRL” group at the before-noon time point serves as the fixed reference. Statistical significance was defined as *P*<.05.

Fixed effect	Estimate	95% CI	*P* value
Intercept (Same and non-BCRL and before-noon)^a^	0.378	0.376 to 0.380	<.001
Same and BCRL and before-noon	0.002	–0.002 to 0.06	.28
Different and non-BCRL and before-noon	0.003	0.000 to 0.006	.047
Different and BCRL and before-noon	0.003	–0.000 to 0.007	.07
Same and non-BCRL and after-noon	–0.001	–0.001 to –0.001	<.001
Same and BCRL and after-noon	–0.002	–0.002 to –0.001	<.001
Different and non-BCRL and after-noon	0.000	0.000 to 0.001	<.001
Different and BCRL and after-noon	–0.000	–0.000 to 0.000	.32
Random effect (group variance)	0.000	—^b^	<.001

a Intercept: reference group representing participants with the dominant arm affected and consistent stage 0 status, measured during the before-noon period.

b Not applicable.

At the before-noon time point, the “different and non-BCRL” group showed a significant increase of approximately 0.003 compared with baseline (*P*=.047). The analysis of the interaction between time of day and group status revealed distinct diurnal patterns. Notably, the “same and BCRL” group exhibited a significantly greater reduction in the affected arm ECW/TBW ratio in the afternoon (interaction coefficient=−0.002; *P*<.001) than the baseline group. In contrast, the “different and non-BCRL” group reflected a significantly smaller afternoon decrease (interaction coefficient=−0.000; *P*<.001).

Overall, while absolute differences in mean ECW/TBW ratios across groups were relatively small, diurnal variation patterns differed significantly. The same and BCRL group exhibited the largest magnitude of afternoon decrease, suggesting that diurnal changes are closely associated with clinical BCRL status and daily loading of the dominant limb. The minimal random intercept variance indicates that variability was more strongly explained by within-individual factors—such as measurement timing—than by between-individual differences. This underscores the need for standardizing home-based measurements to a fixed morning time to ensure data interpretability and minimize inherent physiological noise in longitudinal self-monitoring strategies.

## Discussion

### Principal Findings

This study evaluated the technical agreement between in-clinic and home-based bioimpedance systems and characterized the longitudinal and diurnal patterns of segmental ECW/TBW ratios over 12 months. Our findings demonstrated that home-based BIA measurements using the BWA ON device are highly consistent with professional in-clinic standards (IB770), supporting the reliability of patient-led remote monitoring. However, longitudinal analysis revealed that ISL stage transitions do not always follow uniform BIA directional patterns, highlighting the inherent physiological heterogeneity within clinical stages. Notably, the linear mixed-effects model identified that measurement timing (diurnal variation) is a more significant driver of ECW/TBW variability than interindividual differences.

### Technical Agreement and Feasibility of Patient-Led Monitoring

The strong agreement observed across all body composition parameters (Table 3), particularly the high ICC and CCC values for segmental ECW/TBW ratios, underscores the technical validity of home-based BIA. Despite the lack of clinical supervision, participants demonstrated sustained engagement, contributing over 16,000 measurements with a 64.3% adherence rate. This high volume of real-world data suggests that patient-led monitoring is not only feasible but also provides a much denser data stream than intermittent clinic visits, which are typically limited to 3-month intervals.

### Interpretation of Diurnal Variations and Limb Dominance

A key contribution of this study is the characterization of diurnal ECW/TBW fluctuations. While absolute differences between groups were small, the same and BCRL group (dominant arm affected with a history of BCRL) exhibited the most pronounced afternoon decrease (Table 5). This suggests that the dominant limb, which typically undergoes higher daily mechanical loading, may experience more dynamic fluid shifts in individuals with a history of lymphatic impairment. The near-zero group variance observed in our mixed-effects model further confirms that these fluctuations are primarily a function of measurement timing rather than inherent differences between individuals. Consequently, interpreting a single ECW/TBW value without considering the time of day may lead to clinical misinterpretation of a patient’s edema status.

### Clinical Implications for Remote Monitoring Protocols

Our results provide a practical evidence-based framework for standardizing home-based surveillance. Given that sustained twice-daily measurements may be logistically challenging for long-term self-care, we propose that home-based monitoring be standardized to a fixed morning time–ideally within 1 hour of waking and prior to daily physical activities. This standardization minimizes physiological “noise” caused by diurnal loading and ensures that longitudinal trends reflect true changes in lymphatic health rather than transient daily fluctuations.

### Limitations and Future Work

This study has several limitations, including a relatively small sample size, which may limit the generalizability of the diurnal patterns across broader populations. Additionally, the 3-month interval for clinical ISL staging restricted our ability to correlate high-frequency BIA data with immediate clinical changes. Future research should use more frequent clinical assessments or digital symptom tracking to further validate the sensitivity of home-based BIA trends in capturing subclinical fluctuations.

### Conclusion

In conclusion, home-based segmental bioimpedance analysis provides a reliable and technically valid method for longitudinal lymphedema surveillance. The significant impact of diurnal variation and limb dominance on ECW/TBW ratios highlights the necessity of standardized measurement protocols in remote health settings. By establishing consistent morning measurement routines, patient-led monitoring can provide granular, high-quality data that complement traditional in-clinic care, supporting more precise and personalized management for breast cancer survivors at risk for lymphedema.

## Abbreviations

ALND: axillary lymph node dissection
BCRL: breast cancer–related lymphedema
BIA: bioelectrical impedance analysis
CCC: concordance correlation coefficient
ECW: extracellular water
IB770: InBody 770
ICC: intraclass correlation coefficient
ISL: International Society of Lymphology
LMM: linear mixed-effects model
TBW: total body water
